# Ordinary defensive medicine: in the shadows of general practitioners’ postures toward (over-)medicalisation

**DOI:** 10.1186/s13010-024-00160-0

**Published:** 2024-07-16

**Authors:** Michaël Cordey, Sophia Chatelard, Daniel Widmer, Patrick Ouvrard, Lilli Herzig

**Affiliations:** 1 Institute of Social Sciences, University of Lausanne, Lausanne, Switzerland; 2 Department of Family Medicine, General Medicine and Public Health Centre, University of Lausanne, Lausanne, Switzerland; 3 French College of General Practice, European Union of General Practitioners, Angers, France

**Keywords:** Medicalisation, Over-medicalisation, Vulnerability, Reflectivity, Primary care

## Abstract

This paper draws on qualitative research using focus groups involving 38 general practitioners (GPs). It explores their attitudes and feelings about (over-)medicalisation. Our main findings were that GPs had a complex representation of (over-)medicalisation, composed of many professional, social, technological, economic and relational issues. This representation led GPs to feel uncomfortable. They felt pressure from all sides, which led them to question their social roles and responsibilities. We identified four main GP-driven proposals to deal with (over-)medicalisation: (1) focusing on the communication in doctor–patient relationships; (2) grounding practices in evidence-based medicine; (3) relying on clinical skills, experience and intuition; and (4) promoting training, leadership bodies and social movements. Drawing on these proposals, we identify and discuss five paradigms that underpin GPs’ attitudes toward (over-)medicalisation: underlying social factors, preventing medicalisation, managing uncertainties, sharing medical decision-making and thinking about care as a rationale. We suggest that these paradigms constitute a defensive posture against GPs’ uncomfortable feelings. All five defensive paradigms were identified in our focus groups, echoing contemporary political debates on public health. This non-exhaustive framework forms the outline of what we call *ordinary defensive medicine*. GPs’ uncomfortable feelings are the origin of their defensive solutions and the manifestation of their vulnerability. This professional vulnerability can be shared with the patient’s vulnerability. In our view, this creates an opportunity to rediscover patient–doctor relationships and examine patients’ and doctors’ vulnerabilities together.

“There are many cases in which—though the signs of a confusion of tongues between the patient and his doctor are painfully present—there is apparently no open controversy. Some of these cases demonstrate the working of two other, often interlinked, factors. One is the patient’s increasing anxiety and despair, resulting in more and more fervently clamouring demands for help. Often the doctor’s response is guilt feelings and despair that his most conscientious, most carefully devised examinations do not seem to throw real light on the patient’s “illness”, that his most erudite, most modern, most circumspect therapy does not bring real relief.” (Balint M. The Doctor, His Patient and the Illness. New York: International Universities; 2005. [1957].)

“Theories about care put an unprecedented emphasis on vulnerability—taking up that challenge to transform what really counts in today’s hospitals implies letting colleagues inside previously closely guarded professional boundaries” (2, our translation).

## Introduction

Medicalisation can be defined “as the process by which some aspects of human life come to be considered as medical problems, whereas before they were not considered pathological” [1]. From a Foucauldian perspective, medicalisation can thus be understood as the sociohistorical process in which medical, political, economic, legal and ethical concepts and rationalities began to intertwine in the nineteenth century and which encouraged medicine to be involved in every aspect of life, from medically-assisted reproduction long before birth, children’s education, sports, sexuality and work issues to mourning, sometimes long after death [2–6]. In highly developed countries, the tendency to medicalise ever more aspects of life not only lead to the overuse of medical resources but also to more societal problems and individual illnesses that health care systems and professionals must take care of. Birth, education, work, food and every aspect of human life and death can be the subject of a medical consultation. Thus, even if modern medicine has contributed to longer and, in many cases, better lives, it has also induced new medical problems, ethical issues, ways of suffering and dying, and new societal responsibilities for health care professionals [7, 8].

Medicine and health care have consequently become ever more intertwined with economic, political and societal issues, especially health care professionals’ roles and responsibilities regarding medicalisation. Professionals face growing pressure about their choices of what should be tested and treated and when and how to orient patients. Indeed, debates on these topics arise within and between different categories of health care professionals, putting pressure on them to take up or build a philosophical posture on medicalisation. By posture, we mean the lasting associations between mind and body, thoughts and behavior, and discourse and ways of being in the world. Because the concept of *representation* is mainly treated as an abstraction and the concept of *point of view* is mainly used non-relationally, we opted for the concept of *posture* to emphasise the ethical, very concrete and performative stance that people express in their daily lives filled with ideas, concepts and language [9, 10]. In that sense, the debates surrounding medicalisation have also allowed general practitioners (GPs) to proclaim their unique identity and define their roles and responsibilities towards patients and health care systems. Moreover, in the last few decades, movements such as “less is more” and “choosing wisely” have participated in nurturing social awareness and mobilizing people against medicalisation [11]. Among their numerous theories, the modern world’s reliance on economic interests led to evidence-based medicine (EBM) and the commodification of our bodies and health care, both subjected to market forces [12]. This leads to discussions about good and bad forms of medicalisation and then to the concept of *over-medicalisation* [13, 14].

According to the literature, there is a strong relationship between medicalisation and over-medicalisation. Indeed, each time the issue of medicalisation is raised, it is associated with questions about whether there is too much—whether there is not enough is barely ever discussed. Nevertheless, no common definition of over-medicalisation exists. In fact, over-medicalisation is a conceptual nebula that includes misinformation, disease-mongering, over-screening, over-diagnosis or over-treatment.

### Engaging with the normative underpinnings of GPs’ postures toward (over-)medicalisation

When a patient consults a GP about a complaint or symptom, their decisions whether to explore, treat, act or not act require choices that may put them in a dilemma. Rather than a priori framing the issues in terms of choices and dilemmas, we wish to draw attention to the normative way GPs address issues of over-medicalisation. As we repeatedly refer to those closely associated concepts, we decided to use ‘(over-)medicalisation’ as an abbreviation for ‘medicalisation and over-medicalisation’. We also wanted to reflect upon the conceptual networks and rationalities underpinning GPs’ experiences, feelings and claims about (over-)medicalization. We especially wanted to engage with the topics that matter to GPs when they speak about (over-)medicalisation. Due to the anthropological and inductive posture adopted in this paper, rather than covering GPs’ viewpoints with abstract definitions, we focus on how they experience, speak about and define issues surrounding (over-)medicalisation. Do GPs make any clear-cut distinctions between medicalisation and over-medicalisation? If not, what are the distinctions that matter to them when they talk about those two concepts separately?

Of course, GPs’ uses of those terms could be informed by definitions, concepts, theories and ideologies that are widely spread across society. Indeed, (over-)medicalisation interests researchers in ethics, philosophy and the social sciences as well as physicians, patients, patients’ associations and citizens more broadly [15–17]. Considering this, we wish to focus on two ways of thinking of and engaging with (over-)medicalisation: *rational* and *reflective*. Both ways are important because they provide a framework within which to think about attitudes to and claims about (over-)medicalisation.

*Rational thinking* focuses on developing knowledge, criteria and defined conceptual frameworks about (over-) medicalisation. For instance, when trying to define concepts, ethicists build theoretical models based on predefined values, whereas epidemiologists and physicians try to define criteria and thresholds to distinguish good and bad medicalisation [14, 18]. In this utilitarian way of thinking, the pragmatic presupposition is that knowledge and well-defined concepts will help health professionals and patients to judge clinical situations, agree with each other and make reasoned legitimate decisions. In modern medicine, this rationalist, pragmatic ideal is embodied by the EBM model, associating the best scientific knowledge, as formulated in clinical guidelines, with GPs’ experiences and patients’ preferences to reach reasoned, legitimate, clear-cut agreements through moral reasoning and shared decision-making [19–22].

By contrast, *reflective thinking*, which was particularly inspired by critical reflective anthropology and promoted by Schön [23] in medical practice, asks how medicalisation is experienced, lived, thought about and put into practice in people’s everyday lives. Rather than promoting an idealistic, problem-solving philosophy in medicine, the reflective posture claims, more humbly, to highlight our anthropological difficulty in acknowledging our ordinary, normative and limited ways of sensing, speaking, thinking and being in the world [24]. In a nutshell, this means that, as human beings, we have difficulties recognising—and we usually fail to recognise—the fact that our attitudes, practices, voices, claims and experiences are habitual, unconscious, normative and bounded [25–27]. Thus, a reflective posture suggests that we “descend into the ordinary” to shed light on the opacity of our everyday practices—on what usually stays hidden in the shadows of the ordinary [28]. Here, our anthropological perspective draws on Wittgenstein [29], who wrote that “the aspects of things that are most important for us are hidden because of their simplicity and familiarity. (One is unable to notice something—because it is always before one’s eyes).” In other words, a reflective posture seeks to shed light on things that are so close, usual and intimately linked to ourselves that we do not see them [30], on our difficulty in seeing the visible [31] and on addressing the effects, consequences and issues of that difficulty.

Considering this introduction, we do not plan to provide any clear-cut definitions of medicalisation and over-medicalisation but instead reflect on what is at stake in GPs’ postures toward those concepts. To explore this, this paper focuses mainly on the common ways GPs frame and address the issues surrounding (over-)medicalisation. We asked them to what extent they recognized the limits to their discourse on (over-)medicalisation. In this sense, being reflective does not mean developing a critique of (over-)medicalisation (as it is an entity that could be criticized *in and of itself*); it means investigating the criticisms that GPs may have of (over-)medicalisation, but with one concern: unveiling that part of their criticism that is being left in the shadows.

## Method

This health research project about (over-)medicalisation, associating GPs and anthropologists, started in 2013. The cornerstone of our research group was the will to be reflective about normativity within medical practices and medical research. Thus, we conducted qualitative research using focus groups (FGs) to explore GPs’ conceptions, attitudes and representations towards (over-)medicalisation. More precisely, we wished to study how GPs described what mattered to them in terms of (over-)medicalisation. What were they concerned about? What kinds of problems could they identify? What was troubling them? What kinds of difficulties were they facing, and how did they feel about them?

### Focus groups and participants’ characteristics

Using *convenience sampling*, we took advantage of GPs’ continuing medical education (CME) events to organize seven FGs between January and March 2016 [32]. Each FG started with two very general questions: “What do the concepts of too much or not enough medicine bring to mind in you?” and “What are the determinants of whether or not you medicalise the issues patients bring up in your consultations?” FG facilitators rarely had to rekindle participants’ interest or reframe the discussion, and we did not want to. We were much more interested in knowing how the GPs themselves would set boundaries to the terms of their debates; letting them speak freely about (over-)medicalisation would reveal the topics and issues GPs were concerned about.

Despite our *convenience* sampling method, we made an effort to maintain participant diversity regarding age, practice location and potential involvement in larger institutions (e.g. a university, continuous learning association or political group). Attention was also given to selecting GPs with different degrees of knowledge or experience in the human sciences. Thirty-eight GPs aged from 25 to 69 years old participated in seven FGs (Table 1). Some were still post-graduates, whereas the oldest ones had been practicing for more than 35 years or were recently retired. They worked in rural or urban settings, alone, with colleagues or in multi-disciplinary teams. Most were of French or Swiss nationality, and the others came from European countries. These countries are characterized by different health systems and payment systems for GPs.

**Table 1:** Focus group characteristics

FG	Length (min)	Participants	Male/Female	Age	Date	Context	Setting	Facilitator	Observers
1	84	8	6 M/2F	42 to 69	December 2015 to January 2016	A group of GPs participating in a CME event organised by a medical association.	A hotel room or restaurant; noisy, warm environments but great interest and focus from participants.	SC	DW & PO
2	80	9	6 M/3F					SC	DW & PO
3	71	8	6 M/2F					SC	DW & PO
4	71	5	3 M/2F	30 to 58	March 2016	A peer group of GPs who meet once a month to discuss their patients.	In one of the participants’ offices. Friendliness.	SC	/
5	36	9	7 M/2F	25 to 66	April 2016	GPs attending a workshop at an international congress.	Three groups in the same seminar room. A relaxed atmosphere with many jokes.	DW	SC
6	36	7	1 M/6F	27 to 44				PO	SC
7	36	8	4 M/4F	29 to 63				LH	SC

The first three FGs were conducted with the same group of nine French and French-speaking Swiss GPs during a CME event. Participants were aware that three researchers (SC, PO, DW) would sit in with them. The event was organized by PO, head of the anthropological section of a French medical CME association. One French GP refused to participate, and another agreed but attended only one of the three FGs. After a first analysis, the research team decided that supplementary GPs were needed to diversify the points of view. A fourth focus group was then conducted within a formal peer group of GPs (FG4). SC, who was part of this group, facilitated this FP. The last three FGs were conducted during a workshop at a general practice congress. SC presented the study, and participants agreed to take part in small groups and discuss the question: “What are the determinants of whether or not you medicalise the issues patients bring up in your consultations?” Two of these FGs were conducted in English, and the third in French. SC was present during all seven FGs and facilitated FG1 to FG4. FG5 to FG7 were facilitated by other research team members (DW, PO, LH). An observer chosen by the research staff attended all the FGs except one (Table 1).

### Data analysis

Each focus group was audio-recorded and transcribed, except for FG2, due to a technical problem in the recording. Some analysis of FG2 was done from the observers’ notes. After transcription, we performed a thematic analysis, coding the data using MAXQDA 12 software and classifying them into categories that allowed us to identify the main topics [33, 34]. As defined by Strauss and Corbin [35], we proceeded using an “open coding” approach—inductively examining, coding and categorizing the dataset. SC and DW coded the first FGs independently, as data collection proceeded, and then compared their coding and analysis. The other FGs were coded simultaneously by the same researchers, allowing them to discuss codes and results and produce more analytical *memos*, assisted by the MAXQDA 12 software [36]. SC and DW presented their results to LH, allowing her to give feedback and enhance the final results. All the results shown come from GPs’ discussions during the FGs. Anthropologists MC did not take part in producing the dataset; however, the transcriptions, coding and results were shared and discussed with him at regular meetings.

## Results

### GPs identified (over-)medicalisation in most medical fields

**Fig. 1:**
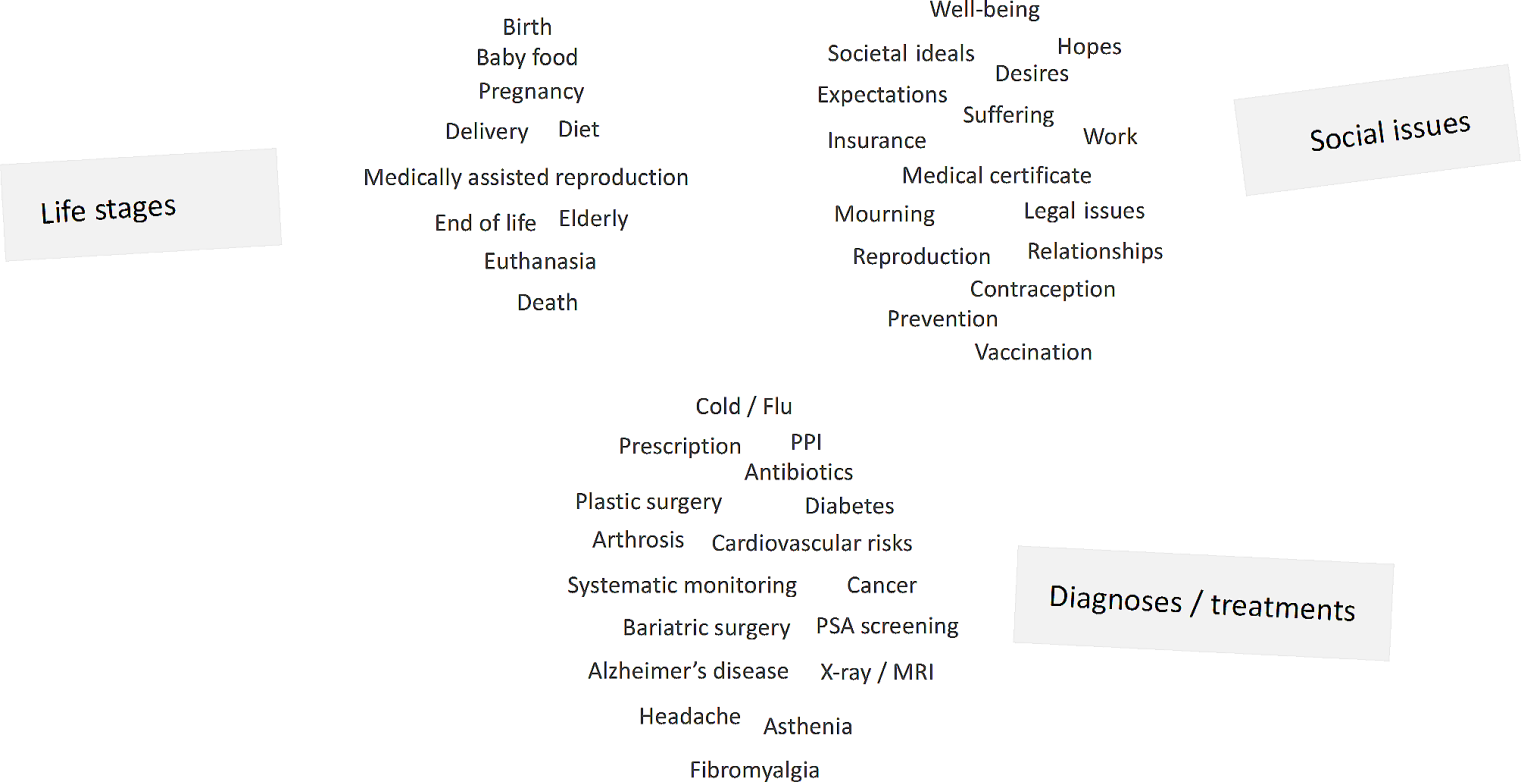
Topics covered by participants

When asked about (over-)medicalisation, participants immediately began to present examples involving numerous medical and non-medical conditions. They cited specific practical situations involving patients with very common or very serious conditions, mentioned issues considered to be more social or societal than medical, and discussed attitudes to different stages of life, diagnoses and treatments. The borders and definitions of fields of discussion were not always clear, as illustrated in the word cloud in Fig. 1.

### GPs tried to define the boundary between medicalisation and over-medicalisation

On the one hand, the GPs interviewed defined medicalisation by the patient’s decision to ask for a medical appointment: “It’s impossible not to medicalise if patients come to see you.” (FG7). It could also be defined by the patient’s point of view: “When someone says they have something, then they have something!” (FG5) On the other hand, GPs also defined medicalisation by GPs’ answers to patients’ demands. They defined different phases in the consultation, including a non-medical part: “the part where I do the physician thing, and the moment where I take time to talk with patients about other things” (FG7). Another GP said that “giving advice is not medicalisation.” (FG5) But others argued that social interactions with a patient could be therapeutic. To overcome this dividing line, one distinguished between prescribing and prescription-free care: “When I don’t prescribe anything, that’s not medicalising. But I’m still taking care of things.” (FG5).

While trying to define the boundary between (reasoned) medicalisation and over-medicalisation, GPs revealed several ideas and examples and asked many questions. They proposed some hypotheses based on their experiences, and discussed their relevance, but failed to find an appropriate definition for that boundary. Under-medicalisation was only discussed in FG4, leading those GPs to talk about patients’ observance to clinical preventive examination and treatments, and especially the fact that patients do not always follow their recommendations, which is considered as under-medicalisation for some GPs.

GPs’ FGs discussions included the following questions and hypotheses. When does medicalisation begin? When someone attend an appointment? Is (over-)medicalisation a question of choice—of acting or not acting? Is it a question of doing more harm than good, or is it one of being able to justify a medical choice? Does it happen because of a wish to avoid risks? Is it a problem of impatience? Should we medicalise social well-being? Is patient education already (over-)medicalisation? Is a patient’s choice to treat themselves (self-medication) (over-)medicalisation? Is continuing clinical investigations until something is found (over-)medicalisation? At the end of a consultation, is prescribing tests or drugs (curing—making the doctor feel like a doctor) done to the detriment of listening and caring (caring—making the doctor feel like a human being)?

### GPs questioned their role and that of medicine

GPs questioned their role, and the role of medicine in general, in different ways. Is their role only to avoid patient suffering, or is it to make patients healthy again? Is it to modify the quality of life or even to “improve the human condition”? (FG1) Do GPs have a spiritual influence? “Before there was religion, now it is medicine”, argued one GP. (FG1) Moreover, should medicine have to deal with welfare and social issues?

The answers to these questions indicated the many ways in which GPs put (over-)medicalisation into perspective, reflecting the many ordinary situations they have to deal with in their daily practice. One GP considered that, at a minimum, she was obliged to guide people towards appropriate professionals, e.g. a homeless refugee who came for a medical certificate: “(…) it’s also to do with this privileged position in a network. So, when someone comes to you like that, you can’t find them a house, but you may be able to find someone who could help them.” (FG7) In this case, she also felt she had a political role: “We could say that physicians, in general, have a responsibility to tell the government that there should be housing for refugees.” (FG7).

### GPs felt pressure from all sides

As GPs tried to identify the determinants of (over-)medicalisation, they revealed strong feelings of having to ‘endure’ over-medicalisation due to many factors that were beyond their control. Over-medicalisation reflected pressure from the outside. “Well, over-medicalisation is not our fault (…) I rather think that we are subjected to it.” (FG1) They spoke about three different kinds of pressures: from society, from the health care system itself and from individual patients’ treatment-seeking behaviours.

### Pressure from society

GPs criticised our society as dominated by consumerism and individualism. “The loss of a sense of community, forced individualism, individual guilt—all those things make us suffer, and our patients too.” (FG3) GPs believed that consumption was responsible for certain mounting health problems, like metabolic diseases and damage caused by environmental hazards. This was put into perspective by some GPs reflecting that their mission was no longer just caring about people’s suffering and death, but also now about their “quality of life.” (FG1).

Pharmaceutical companies’ roles in this were pointed out, and the media were accused of promoting the system and ideals of physical appearance and lifestyle. “The trend that makes people (…) aspire to have no pain, no wrinkles, no hair falling out, no belly fat, and things like that.” (FG3) One GP noted that “We’re asking people to adapt themselves to their changing social environment that produces pathologies caused by our way of life.” (FG1) For instance, another argued, “There’s all this aspect of having an ideal body, and the development of coaching that implies that everyone needs a heart-rate monitor when going running. People also want to measure their ideal weight and their adipose mass. All that’s to say that one can see that medical concepts, [ideas and methodology] are invading” our daily lives. (FG1)

Another reason explaining over-medicalisation was a fear of lawsuits. For instance, one GP said, “I’m currently working in an emergency ward, where I know that I won’t be seeing this patient again, but they’ll remember me, and they’ll remember my name on a piece of paper or a letter. And when I didn’t want to prescribe X-rays or medication, my senior said to me, ‘From a legal point of view, do it.’ Nobody wants to take the risk these days.” (FG5).

### Pressure from the health care system

GPs discussed three main ways the health care system put pressure on their daily practice: (1) the political economy of health care; (2) the request for administrative assessment; and (3) the power of prescription.

1) When considering the political economy of health care, GPs mentioned that physicians in France worked on a fee-for-service system. Several GPs thought this a “perverse” system, leading to over-medicalisation for profit. They thought that perhaps a flat fee per patient would make GPs less prone to over-medicalise. One noted, “Obviously if you paid the full price for each test, I mean, you would not get yourself X-rayed for everything and anything.” (FG4) On this point, “Everything is defined by the social security [system],” said one GP, while arguing that less invasive therapies, like psychotherapy or psychological care, did not fall under the domain of the social services. (FG1) Considering this, one GP argued, “Over-medicalisation depends on a country’s wealth. That’s to say, what is covered by [health] insurance and, thus, a country’s political economy.” (FG4).

2) Because of their role as medical experts within the health care system, GPs have to perform many kinds of administrative medical assessments. As one GP in France described it, “Typically, people who are in distress and who, after having consulted a social worker, go to a physician to ask for a certificate for the MDPH (French Departmental Home for Disabled Persons).” That certificate ‘qualifies’ people as disabled and gives them access to MDPHs where they can be informed, oriented and get a disability allowance. (FG1) Other administrative assessments mentioned were sick leave certificates for school and bank loans. One GP mentioned that “In some countries […] you have to go to a physician first to get access to further support.” (FG7) Another argued that this showed how “Social issues are overwhelming medicine with topics that do not concern it.” (FG1).

3) One GP was concerned about the power that prescriptions have in the doctor–patient relationship. According to him, that power depended on the health care system’s political and economic structure, which defined who had the right to deliver medicines and which roles different types of professionals could play within the health care system. He was worried about the fact that “the less we prescribe, the more we are losing our power to do it, the more pharmacists gain from it.” (FG4).

### Pressure from patients

GPs felt difficulties dealing with those patients who wanted everything, easily and immediately. People just want “to be cured very quickly—everything has to be quick—and they don’t understand, for example, that for flu, you need to wait.” (FG7).

One GP’s explanation for patients demanding medication and investigations was their absolute belief in science and technology. “People’s belief in the power of technology can lead to over-medicalisation.” (FG7) Another explanation for their insistence on being treated was their faith in the media. “There’s the women’s press, the mainstream press, and there’s the internet and internet forums where you can find a miracle solution.” (FG1).

Only one GP thought that medicalisation was justified most of the time and thus that over-medicalisation was not an issue. He believed that patients and society were benefitting from technology and science and that it was a GP’s responsibility to use those to do everything in their power to prevent disease: “We cannot afford to miss something that is detectable.” (FG3) His reasoning was that if it was a GP’s responsibility to catch every detectable disease, then responsibility, medical reasoning and decision making had to be delegated to science to avoid care turning into chaos: “I think that regarding therapeutics, physician bend—in quotation marks—to the guidelines that come out. We have to rely on something concrete. If we couldn’t, then I would say there would be a total breakdown.” (FG3).

### GPs felt uncomfortable

#### Feelings of guilt

Echoing the reasoning expressed above, GPs expressed significant feelings of responsibility and guilt regarding (over-)medicalisation. They thought that medicalisation could be a way to keep power and to “continue to reinforce” their status. (FG4) Regarding patients, GPs also criticised that patients asked them to be “omnipotent” and proactive. One GP said he felt “the urge to give something, even if it was a placebo.” (FG7) Thus, GPs recognised that their actions might be induced by their own fears. As one argued, “I think physicians also have their own anxieties that prevent them from resisting this movement as much as they could.” (FG3).

### Fearing regrets: anticipation, patients’ desires and uncertainty

When analysing specific cases, GPs knew that they could really only decide whether their clinical decisions had been right *a posteriori*. But there were two ways things could have gone wrong: having done too much (e.g. by extending a sick leave, with negative consequences on a patient’s ability to return to work) or not having done enough (e.g. not having convinced a patient to continue her hormone therapy when she developed metastases years later). “I should perhaps have insisted that she took it.” (FG4).

GPs’ difficulties in finding the right balance between not enough and too much were not only a question of judgment and action but also of dealing with anticipation and uncertainty. One GP described a patient who came to his surgery because he was having memory troubles and the two attitudes, he had toward this. The initial one was to say that everything was all right and that it was normal to forget a few things at 85 years old. But difficulties arose when the GP realised that his patient lived alone, had comorbidities and had to take drugs daily. What if he forgot his medicine or left the cooker on? Thus, the secondary attitude was to anticipate a much more global way of caring for patients, but one that might go against their wishes. Here, the tension, pressure and discomfort felt by GPs was because they were anticipating social and moral issues and because they felt responsible for dealing with so many uncertainties. (FG4)

One GP described how, in training, they had learnt to think systematically so as not to miss anything and find the right diagnosis, but they had never been trained to live with uncertainty. (FG6) Thus, clinical examination and the quest for diagnoses were sometimes seen as the normal answer to medical uncertainties because of over-medicalisation and GPs’ fears of missing something they would later regret. Considering this, GPs acknowledged their fears and their potential for improvement. As one GP remarked, “We must learn to manage our uncertainties.” (FG6).

### Feeding anxiety and creating a dependency on biomedical solutions

Some GPs said they felt discomfort about their attitude towards clinical examinations and the quest for diagnoses. They knew this might produce anxiety and feed worries, which was, in their opinion, especially useless when clinical examination and the quest for diagnoses sought diseases for which there were no therapies. Moreover, one GP argued, “When repeating clinical examinations, we will necessarily find something.” (FG4).

One GP described his clinical experience of this with an infant and their parents. The infant presented with gastroenteritis, and the physician asked for a blood test, which, he said, he nearly never did. Because the test revealed leukopenia, this led to an overall assessment including a myelogram, which is very invasive and produced a lot of anxiety, especially for the infant’s parents. In the end, the GP took the initiative to tell the parents to stop all the monitoring and clinical examinations because the infant was doing very well and the situation was just producing stress for no reason. The situation, he said, “had become deleterious for everyone.” (FG4).

Prescribing medicine, just like prescribing a clinical examination, was also a source of and a means of responding to physicians’ and patients’ feelings of discomfort. Indeed, GPs were aware that prescribing medicine was a powerful way of managing not only their patients’ anxieties but their own too. Some GPs argued that patients and GPs needed to be educated about this. The issue, as one GP said, “is that it is easier to prescribe than to find a way to change social health behaviour.”(FG6).

On that topic, another GP described a homeless man presenting with chronic bronchitis and asthma who went to the hospital repeatedly. Physicians there talked about immune deficiency without diagnosing anything. For the next ten years, every time he caught a cold, the homeless man came to see the GP because he was afraid. The issue, for this GP, was that it could take a long time for people to do without biomedicine and physicians. (FG4) Another GP argued that the issue was about explaining and educating patients because, he said, then they would be “more serene about their problems”. “You explain things”, he argued, “and the next time, they know how to do it alone.” (FG4).

### Proposals for dealing with (over-)medicalisation

GPs laid out several different things they could do to avoid (over-)medicalisation, focusing on four main dimensions: (1) communication in the doctor–patient relationship; (2) scientific knowledge, technologies and evidence-based rationality; (3) clinical skills and reflexivity; and (4) activities outside the consulting room such as involvement in teaching, research, professional trades unions or political activities.

### Focusing on communication in the doctor–patient relationship

GPs viewed a close doctor–patient relationship as the best way to avoid (over-)medicalisation. As one argued, “I feel that we really have a role to play because we really know the patients and their problems. […] We can offer the patient a rather holistic type of care, that’s to say, really using all our skills as a GP.” (FG4) Table 2 includes some examples of dialogue from the many moments when GPs discussed their skills.

**Table 2:** Quotations illustrating GPs communication skills

Listening to the patient	“It’s not so much the fact that they know how to manage the cold, I mean, but it is rather that we listened to them. We also heard the complaint that was behind it all.” (FG4)
**Not blaming the patient**	“We spoke. I gave him advice, not treatment. And after two months, he had almost normal blood sugar. And he was very happy. He didn’t feel guilty; he just understood…” “Yes, but you didn’t just say, ‘You eat too much; you’re too big; you’re not doing enough sports.’ ” (FG3)
**Speaking with the patient**	“For me, speaking, communicating and being in a relationship are fundamental elements in reducing drug consumption.” (FG3)
Exploring patients’ representations, feelings and experiences	“It might be interesting to discuss what it feels like in their heads—to do that in relation to how they see the disease, how they think they are sick.” (FG3)
Finding patients’ hidden agendas	While speaking about diabetes, one GP talked about the idea of “working on how one should live at home; how one should do the cooking; what the values of different foods are; how one represents eating this or that.” (FG3) “A cold is sometimes only the hook, and then once that’s all right, you can start to understand what the real reason behind the consultation is.” (FG4)
Explaining, taking the time and educating the patient	“We have to explain to the patient that, ‘Yes, you have a disease, but if you wait, sometimes there is no problem at all.’ ” (FG5) One GP argued that after having explained things and educated his patients, “They are more relaxed about their problems […] and the next time, they know how to do it alone.” (FG4)
Delivering preventive messages and informing the patient	“It is true that if we are disciplined and take the time, at every consultation, to give out a preventive message every time, it leaves a mark.” (FG1)
**Reassuring the patient**	“When you start to reassure them, you start to reduce their consumption, actually.” (FG3) “I am amazed how often patients are reassured. Very often, they will come to see me with a severe fever, or they are very sick. What they want to hear is that it’s just the flu, and they can go home. Sometimes that can be enough.” (FG7)
**Allying with the patient**	“Our patients are often objective allies, helping to lessen the burden of a number of things, and sometimes to demedicalise things too, yes.” (FG1)
**Exploring patients’ requests**	“We really have to work on finding out what people want.” (FG3)
**Helping patients to find meaning**	“Yes, but to what extent, in your relationship with your patient, can you help him find a meaning to his pain, maybe not in a religious sense, as we used to, but with the idea that you can make sense of his suffering for him. I’m convinced that’s very helpful.” (FG3)

### Grounding practices in evidence-based medicine

For some GPs, some of the recommendations of EBM are unavoidable (e.g. regarding diabetes). None of the GPs was radically or ideologically opposed to EBM, and some even evoked that scientific knowledge helped their decisions not to (over-)medicalise. One argued that, “I get the impression that we can save on a lot of treatments, care, technical acts and more if we integrate scientific evidence.” (FG4) Moreover, although GPs were sceptical of medical practices unsupported by evidence, they did not speak about EBM as a body of rules to be followed blindly; they were aware that they used EBM in consideration of the specificity of particular clinical situations. (FG4, FG3)

Finally, one GP insisted on the power of modern tools, saying, “We have modern tools; we have to use them. For someone suffering from back pain, I would suggest doing a radiography to see if there is a malformation […]. And then you can explain things a bit […], and it could be comforting.” (FG3).

### Relying on clinical skills, experience and intuition

The GPs insisted on the importance of a thorough anamnesis and a careful physical examination. “That’s really our added value over the medicine that can be found on the internet! (laughs) Our clinical examination!” (FG4) Ideally, GPs would employ a systematic approach not to miss any urgent cases, and then also prioritise according to their intuition and past experiences. Sometimes they based their decisions on EBM guidelines, considering the intensity of patients’ complaints, the natural evolution of their disease and the resources available.

GPs also questioned their clinical routines, with one especially saying, “The time and the place and the team with which you work significantly change your way of prescribing or medicalising.” (FG5) Considering this, some cited narrative medicine, psychosomatic medicine or transcultural psychiatry as potential means of avoiding over-medicalisation. (FG3) Finally, some GPs insisted on their need to learn to say “no” to patients, whereas others argued that, sometimes, they needed to restrain their propensity to say “no” to patients too easily.

### Promoting activities outside the consultation such as teaching, research, professional trade union or political actions

GPs criticised initial hospital-based training as too hospital-centred, and they alluded to its complexity and uncertainty. One GP argued, “In the French medical system, you have to pass an exam at the end of the sixth year, which is organised speciality by speciality and, concerning myself, when a patient arrives with a problem or a question that is related to a speciality I know I’m uncomfortable with, I know I have a tendency to … (laugh) prescribe more biology or radiology.” (FG5) Post-graduate training in general practice could remediate this by looking at more complex, real-life clinical situations. As another GP said, “Besides, there was something that shocked me in general medicine courses. You go finished them and I asked myself, ‘What did I attend that for?’ Because you didn’t have any more answers to your questions (laughs)!” (FG6).

Some GPs were aware of the role they could play in facilitating social changes by being more involved in movements concerned with the evolution of general practice. In doing so, they could elaborate their own guidelines, as was done with the “less is more” and “choosing wisely” movements. As one GP argued, “Physicians created these for themselves.” (FG1) Others argued that it was important to take part in ‘quality circles’ in order to exchange clinical experiences between colleagues. (FG1) Another GP emphasised that they should even become involved in public or professional associations to take part in political decisions.

**Fig. 2:**
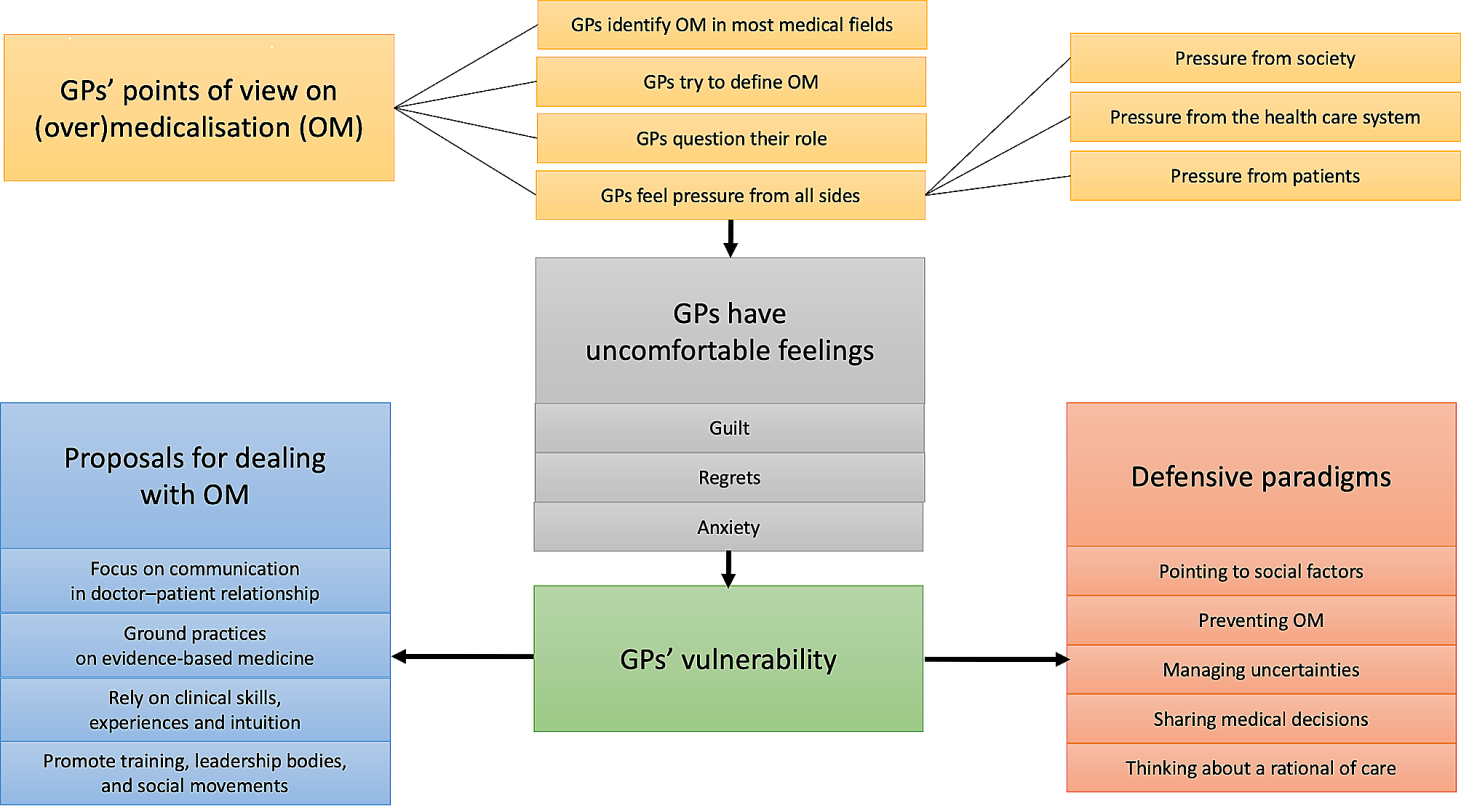
Conceptual map of our findings

## Discussion

Seven FGs involving 38 GPs provided a picture of their attitudes towards (over-)medicalisation. Firstly, GPs were very interested in this topic. Secondly, they had no consensus definition of (over-)medicalisation, nor could they define the boundary between medicalisation and over-medicalisation, between just enough and too much, and we note that under-medicalisation was never discussed. Instead, the FGs revealed a much more complex reality. The picture was composed of several intricate issues summarised in the *conceptual map* above (Fig. 2). Examining this map, we see that how GPs thought about (over-) medicalisation was not reducible to the rational models and concepts promoted through EBM, shared decision-making or patient-centred care, which are today considered the gold standards for medical reasoning. As Fig. 2 reveals, GPs also spoke about their feelings related to their social, political and moral roles and responsibilities. Moreover, their experiences of (over-)medicalisation were composed of many different elements, including communication, context, education, science, clinical practice, rationality, technology, administration and law, as well as pharmaceutical, moral, ethical, economic and political aspects. In a nutshell, GPs talked about (over-)medicalisation in terms of social and relational issues rather than reducing it to medical and rational ones.

Interestingly, our main result showed how uncomfortable GPs felt about (over-)medicalisation. They knew, of course, that managing clinical problems depends primarily on medical knowledge, but they also clearly relate it to social and relational factors. In this way, medical practice is also subject to both “clinical uncertainties” (that may be managed through scientific knowledge) and “existential uncertainties” (depending on social and relational pressure) [37–39]. Considering this, we believe that GPs’ feelings of discomfort are not only related to their uncertainties about their knowledge but also to their fundamental anthropological and sociological vulnerabilities.

Regarding the ethics of care, vulnerability does not refer to the vulnerabilities of a particular population (e.g. children or the poor) but rather to patients’ and doctors’ common human and relational vulnerabilities (of meaning and values, as well as toward misfortune, illness and death) as Paperman argued [40]. She wrote that the significance of *care* “requires recognising that dependence and vulnerability are traits that everybody shares, even if the most fortunate among us have the capacity to soften or deny their intensity”, and they do this by using defensive attitudes.

Figure 2 shows GPs’ attitudes to dealing with their uncomfortable feelings: the left column summarises GPs’ four proposals for managing (over-)medicalisation; the right column summarises what we have identified and called the five defensive paradigms encompassing GPs’ attitudes to (over-)medicalisation. As we will discuss further, management and defensive postures are embedded in GPs’ proposals for coping with their feelings of discomfort. We see those feelings as being linked to GPs’ common vulnerability, as described by Paperman. According to Fassin, GPs today have to ensure that the care they provide patients is *biolegitimate*, which means including patients’ viewpoints and negotiating medical decisions [41]. Our questions here were twofold. Firstly, how much GPs can think their medical practices in terms of dependence and vulnerability? Secondly, do their attitudes toward (over-)medicalisation embrace dependence and vulnerability or deny it? This discussion of our results also tries to link GPs’ proposals about (over-)medicalisation to their techniques for coping with their vulnerabilities.

We will now discuss the five paradigms identified in our FGs: (1) Pointing to social factors; (2) Preventing medicalisation; (3) Managing uncertainties; (4) Sharing medical decision-making; and (5) the Rationale for care. We will try to explore the relationships between those paradigms and what we have called *ordinary defensive medicine*. Also, the discussions sketch out a critique of the postures that GPs adopt towards (over-)medicalisation. Indeed, while speaking about it, GPs showed us different ways of reacting to their vulnerability and the vulnerability of others. Our main criticism of this is that most of the concepts and proposals used to speak about (over-)medicalisation (e.g. communication, clinical skills, EBM or training) were ways to push for more rational management and defensive attitudes rather than towards the acknowledgement that GPs and patients are vulnerable. And this led us to ask ourselves about the politics and ethics of vulnerability in family medicine and medicine more broadly.

### Ways of being defensive

#### Pointing to social factors

The GPs interviewed were aware and thoughtful about how their practices were sociologically embedded in relationships and society. They reminded us that (over-)medicalisation was influenced by consumerism, individualism, the pharmaceutical industry, the ideology of performance, the political economy of the health care system, administrative work and legal rules, and the development of new public management methods and biomedical technologies. They thus proposed the development of a critical mind and a sceptical attitude. These critiques have been shared for decades by some of their colleagues as well as the ethicists, philosophers, historians, sociologists and anthropologists who have warned about the risk of neglecting the role of social determinants in shaping human health behaviour (e.g. race, gender and socio-economic status as well as health system organisation, economic pressures, bureaucratic and administrative procedures, medical knowledge, the promises of technology, and ideology). Briefly, participating GPs, as do social scientists, argued that human health behaviours were social constructs and the inheritance of decades of social thinking, which has become a common way—not to say a reflexive one—to address criticism.

Participating GPs tended to speak about (over-)medicalisation in terms of external social factors and abstract definitions and criteria rather than in terms of an inward-looking “relational ethic” in which (over-)medicalisation was entwined in the doctor–patient relationship [42]. For instance, some GPs were insistent on abstract definitions of where (over-)medicalisation started or what constituted ‘too much’. However, does addressing the issue in terms of abstract definitions, boundaries and thresholds lead to avoiding reflection on the doctor–patient encounter? On the contrary, regarding relational ethics, medicalisation does not refer to any abstract definitions or pure sociohistorical determinants. Instead, it refers to the ordinary anthropological processes through which people form and express their postures towards (over-)medicalisation and the effects it has on people’s relationships and behaviours.

Thus, we do not seek to address the issue of the illegitimacy of GPs’ experiences. Neither do we doubt the truth of their sociological claims. We are interested in their tendency to strictly frame criticism about (over-)medicalisation in terms of abstract, distant and disconnected social issues. Indeed, the limitations of social thinking (and especially constructivism) and social determinants are that they point to external factors and issues; as if what was at stake was taking place somewhere else, beyond GPs’ consulting rooms, reducing them to powerless spectators and witnesses. In other words, we think that pointing to social factors is legitimate and necessary. But this can also leave aside GPs’ existential vulnerability. We suggest that denying one’s vulnerability rather than facing up to it and working on it could lead to a rigid defensive posture. The tragedy of social thinking is that it tends to forget that (over-)medicalisation can be discussed and co-created with patients. Thus, during FGs, everything happened as if GPs were predisposed to speaking about (over-)medicalisation in social terms as if this way of thinking was powerful enough to frame most of the debates.

From this point, we move on to a second way of thinking that underpins GPs’ discourse about (over-)medicalisation: the normative horizon of less medicalisation. We see less medicalisation as another way of conceptually maintaining the medical encounter with patients and human vulnerability at unreachable distances.

#### Preventing medicalisation

Many different postures on preventing medicalisation can be identified in the literature. We start by presenting two different paradigms: Jamoulle’s “quaternary prevention” concept and the “choosing wisely” or “less is more” movements.

Jamoulle defined quaternary prevention as the “action taken to identify a patient or a population at risk of overmedicalization, to protect them from invasive medical interventions and provide for them care procedures which are ethically acceptable” [43, 44]. This concept is based on the distinction between disease (the doctor’s view) and illness (the patient’s view) in the context of a primary care consultation. This basic distinction led Jamoulle to suggest four different modes of prevention: primary (“intervention before disease”), secondary (“avoiding false negatives”), tertiary (“curing and preventing complications”) and quaternary (“avoiding false positives”) prevention [45]. Indeed, from Jamoulle’s perspective, medical ethics was especially lacking a concept to qualify the kind of prevention needed when a patient felt ill but no disease could be diagnosed. This particular situation encouraged Jamoulle to make the doctor–patient encounter and relationship central to his medical philosophy of caring. In his medical philosophy, quaternary prevention should not be misunderstood as an injunction to give up caring or save money—another way to push aside the vulnerabilities of patients and doctors [46]. Rather, quaternary prevention’s goal is to prioritise discussions about individual patients’ situations, preferences and needs in the light of GPs’ experiences and medical science, thus respecting Sackett’s definition of EBM [47].

By contrast, the “choosing wisely” or “less is more” movements describe contexts where investigations or treatments are unnecessary, hoping to limit the overuse of medicines and their economic costs [48–51]. These two movements are specifically based on statistical and population-based information to limit health care costs, useless tests and treatments, and iatrogenic harm. Here, the rationale for EBM seems to follow a box-ticking approach to pursuing the implicit normative and abstract horizon of less medicalisation. In this paradigm, GPs can rely on EBM and partially delegate their responsibilities to it, without having to share their vulnerabilities or engage with their patients’ vulnerabilities.

Martins criticised Jamoulle’s quaternary prevention definition because it is limited to clinical situations in which patients feel ill but no disease can be diagnosed [52]. Martins argued that quaternary prevention should go “beyond preventing overdiagnosis or preventing overtreatment; it includes preventing all types of harm associated with medical interventions” [52]. Martins proposed a revised definition: “quaternary prevention as an action taken to protect individuals (persons/patients) from medical interventions that are likely to cause more harm than good.” In a word, Martins suggested extending non-maleficence to primary, secondary and tertiary prevention [52]. According to Martins, “the concept of quaternary prevention is nothing more than the systematisation of the concept of *primum non nocere* in our modern medical practice” [52].

What if we followed Pellegrino and Thomasma’s suggestion and twisted Martins’ arguments into “the primary obligation that unifies the theory of medical ethics is beneficence” rather than non-maleficence [53, 54]. This would imply shifting from a preventive, defensive medical posture, which aims to prevent (over-)medicalisation, to an open, therapeutic medical posture, whose aim is to help both patients and physicians find the best way of living with or without a disease. In other words, a perspective that works with peoples’ vulnerabilities rather than against them. Jamoulle’s and Martins’ positions differ fundamentally on this point. While Martins insists on a defensive concept (the non-maleficence principle), Jamoulle insists on the patient–doctor relationship, the temporality of care and the changes needed for “the doctor to have a critical look at his/her own activity and influence as potentially harmful for the patient and to question the ethical limits of his/her activities” [44]. The difficult choice for GPs “is to explain the doubt”, which is a way of saying that medical practices have to open up to sharing uncertainties and vulnerability.

Finally, if we consider Launer’s “social constructivist” posture, it is astonishing to see how defensive the discourses and theories surrounding quaternary prevention are [55]. Indeed, those discourses are usually interwoven with concepts like harm, maleficence, burden, over-use, workload, misinformation, disease-mongering, over-screening, over-diagnosis, over-treatment, poor outcomes, risks or wasted resources rather than with those about resilience, beneficence or therapeutic power. However, from Launer’s perspective, “the physician not only hears the cue but also offers the patient a chance to extend her story into what is commonly called the ‘lifeworld’ […] helping the patient take her narrative where it needs to go”, which implies transforming (more or less radically) the patient’s representations of illness, their health behaviour and their life. In contrast to the defensive, constructivist posture we discussed previously (pointing to social factors), Launer’s posture suggests a “social constructionism” that implies being open, generous and disposed to being educated by listening to “stories about illness and health” and helping patients and physicians “reconstruct ones that they find more useful or meaningful” [55, 56]. This would imply putting patients’ and physicians’ habits, visions, representations, values and claims to work.

We must be very clear that we are not calling for a massive disruption to medical care or for medical anarchy. We want to consider the anthropological difficulties encountered by patients and physicians so that we can manage to live with uncertainty in the best way possible. This is a call to seriously address the anthropological issues of therapeutic co-formation rather than merely the sociological issues of coordination [9]. While the anthropological issues focus on the co-formation of voices (that are not necessarily shared and allow for the possibility of disagreements within medical decisions) and the ethical issues of acknowledging each other’s claims and visions, the coordination paradigm focuses on individual capacities, moral reasoning and the rational management of pathologies. What is at stake is not reducible to merely a rational way of managing a pathology and its uncertainties, but, more radically, it includes creating and acknowledging the possibilities, changes and choices that imply finding the best possible way of living with or without a disease. And this means acknowledging vulnerability and working with it in order to elaborate particular forms of medicalisation *with* patients rather than denying it while referring to abstract and moral concepts or norms.

Thus, the questions that GPs should seriously seek to address regarding (over-)medicalisation are: How much debate, negotiation and conflict with their patients can they stand? To what extent can they tolerate going through uncertain therapeutic processes with them? To what extent are they ready to acknowledge and accept the vulnerability of their practices?

#### Managing uncertainties

**Fig. 3:**
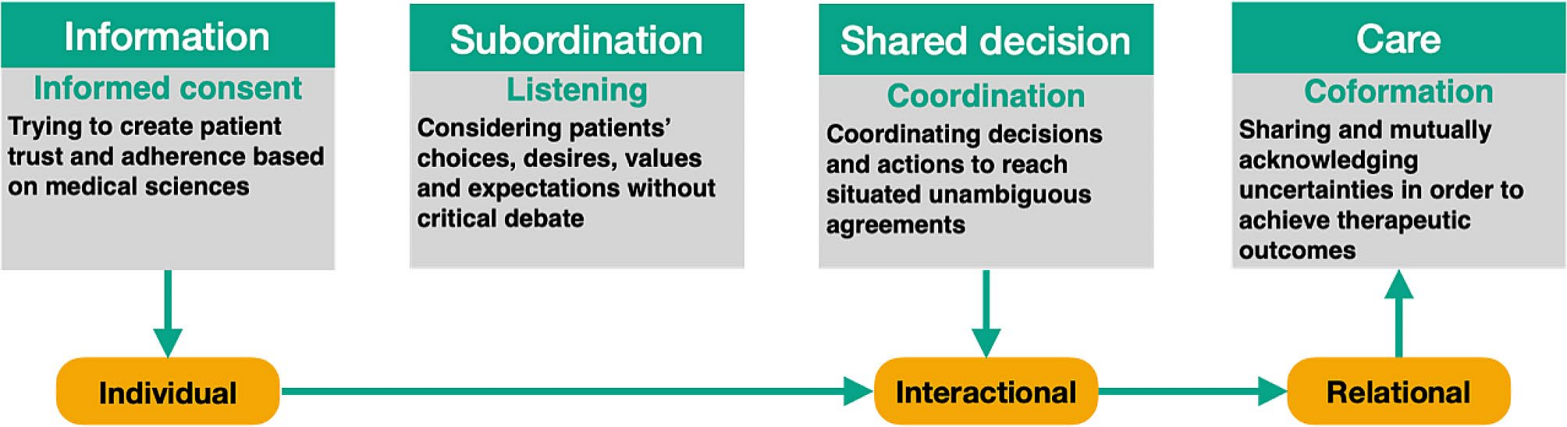
Four medical uncertainty management paradigms

GPs’ discourse about (over-)medicalisation also echoed broader attitudes toward managing uncertainties in medicine. The main stance towards uncertainties in medicine is trying to reduce them or, at least, neutralise them. It focuses primarily on risks rather than on the therapeutic heuristic of uncertainties. Considering this, we argue that the main paradigms for coping with uncertainties in medicine are defensive ones. The different ways of being defensive can be summarised and organised according to four paradigms (see Fig. 3), from the most individual to the most relational one. We will now discuss these paradigms.

According to the *information* paradigm, uncertainties are managed and rationalised through EBM. The ideology is to ground medical judgment and, in so doing, to produce patients’ trust and adherence. The decisional model that reflects this best involves the patient’s informed consent. Examining the literature on quaternary prevention, this model is advocated via arguments such as, “what we need is a strong and sustainable relationship with our patients and their trust in our honesty and specific knowledge” [57]. This implies rational human beings who do not have to share their common vulnerabilities but rather transmit information in order to reach the patient’s decision. Considering vulnerability, this paradigm only focuses on “clinical uncertainties” and not on “existential” ones, which is another way of setting common anthropological vulnerabilities aside.

According to the *subordination* paradigm, the management of uncertainties relies on the crude respect of patients’ wills, desires, values and expectations for their life. No medical decisional model refers to such a non-disruptive attitude. Yet, pushed to the extreme, those paradigms are problematic because they condemn GPs to a paternalistic posture grounded in EBM for the former and to being blindly respectful and passive for the latter [58]. In both cases, neither doctors nor patients are invited to share and acknowledge their vulnerabilities. On this point, our findings showed that GPs did not support these radical postures. Instead, they spoke about the difficulties and tensions that arose within their practices when they had to respect both the will of their patients and EBM guidelines.

Only one GP advocated sticking rigidly to EBM’s rational approach because, he argued, it was the only objective ground GPs could rely on and thus prevent chaos. The other GPs who spoke about this topic had a much more nuanced approach. One of the most cited arguments for not relying blindly on EBM was that physicians can only know *a posteriori* whether their decision was a good one. Thus, they argued, that one can only estimate but not be sure whether a medical decision will be beneficial or harmful in the future. Moreover, there is a recognised strong link between decisions and anticipation of regrets in medicine, which implies that physicians project uncertainties through time [59].

Now, focusing on the defensive concept of coordination, we would like to discuss the paradigms of *shared decision-making* and *care*.

### Sharing medical decision-making

The shared decision-making paradigm is considered the gold standard in medicine [19]. Shared decision-making relies on EBM as it has been defined by Sacket et al. [22]: physicians and patients must discuss their requirements in order to create an adjustable agreement relying both on the rationality of EBM, the therapeutic possibilities available and patients’ wishes, values and expectations for life. In this model, managing uncertainties is often similar to the rationale of coordination. In a nutshell, the paradigm of coordination rests on people’s abilities to justify their decisions in order to agree on suitable actions [60]. Indeed, in the view of those authors, people (doctors as well as patients) can put their judgments and actions to the test while holding them accountable and asking each other for medical and moral justifications. The principle of coordination, which relies on ethical discussion, is widespread in decision-making theory and in public health [61]. Under this rationale, uncertainties are managed within a normative horizon to end disputes and come to an agreement through reasoned processes of justification. Considering this ideology, which relies on well-defined values, ethical principles, reasoned arguments and justification, people should naturally come to realise what matters to them and achieve legitimate, acknowledged agreements.

There are many criticisms of this rational, reasoned and mechanical way of thinking. Firstly, it relies on a narrow, instrumental and structuralist vision of language, which does not reflect the reality of its use in everyday life. It sets aside those particular circumstances and events that give life to our words and expressions [9, 10, 62, 63]. Here, we can speak about coordination as a defensive paradigm because it contributes to denying the vulnerability in our words (that is to say of our ways of thinking, viewing, sensing, valuing, and so on)—the vulnerability that can be expressed in the language of patients and physicians (especially if we pay attention to the tiny details, the tones and rhythms of words and voices) [64]. Moreover, this perspective is idealistic in the sense that it sets aside the fact that medical decisions are not always made by relying on clear-cut, explicit, legitimate agreements; these sometimes happen in a “grey zone of rightness” relying on “equivocal agreement” and creative moral imagination [9, 65].

This led us to ask about how to address the issue of doctor–patient encounters. Should they be seen in terms of achieving a logical, shared, legitimate agreement? Or should they be seen in terms of the difficulties of mutually acknowledging the fragility of each other’s (medical) claims and the vulnerabilities of each other’s visions and voices through sharing experiences, expectations, fears or doubts [64, 66]? While reaching agreements relates to procedural, conventional, rationalised ethics in which moral dilemmas have to be solved by morally competent individuals, vulnerability refers to the immanence of ethics and the co-formation of subjectivities and doctors’ and patients’ voices. In this view, ethics is much more a question of ordinary relational tensions requiring time, attention to people’s concerns, and the expression and acknowledgement of vulnerabilities [9]. In conclusion, if shared decision-making is only thought of as a rational way of managing uncertainties – rather than a way to share vulnerability and acknowledge it –, then it is one other defensive attitude that contribute to maintaining the taboo of vulnerability in medicine.

### The rationale for care

The paradigm of *care* relies on openness in which: (1) the fragility of points of view, visions and claims can be mutually revealed and shared; (2) what really matters to people is explored and expressed collectively; and (3) ethical tensions can find a way to be released through the formation and acknowledgement of each other’s voices. This especially implies that we think about ethics not as a critical moment of truth and coordination but rather as a process of revelation through which people’s voices, subjectivity and sense of importance are co-formed through listening and acknowledging other’s expressions and words. These considerations lead us to further discuss how the paradigm of *care* can be understood as a defensive one.

The *shared decision-making* and *care* paradigms echo Mol’s [67] “logic of care”. Mol developed the logic of care when considering diabetes—a chronic disease that can be partially managed with medical technologies. For Mol, the logic of care was a critical response to the “logic of choice” that broadly prevails in medical ethics, especially within the informed consent model and most shared decision-making theories. Indeed, Mol critiqued the ideology of choice that has prevailed in medicine and that supposes rational, autonomous and competent subjects able to make clear-cut, non-revisable decisions based on well-defined arguments. In contrast to the logic of choice, she argued that in the logic of care, the issue was the continuous adjustment made between the patient’s life and medical possibilities. Thus, according to Mol, *care* is a continuous, open-tuning process relying on an exploration of medical possibilities available and patients’ wishes, values and expectations for their lives.

Yet, although Mol’s logic of care insists on the necessary and experimental adjustments between patients and medical technologies, it does not address the issue of vulnerability. To put it differently, from Mol’s perspective, the issues of medicalisation and vulnerability are reduced to one practical issue—that of finding the best match between the patient’s disease and medical technologies through trial and error. Concerning the management of uncertainties, Mol’s logic of care was more or less reduced to technological and “clinical uncertainties”—i.e. when the limits of necessary knowledge are well defined and bounded—which avoids discussing “existential uncertainties” and putting GPs’ and patients’ vulnerabilities on the Tables (39, 40).

Thus, the prerequisite for Mol’s logic of care is two people who already agree—more or less precisely, on medicalisation and medical promises—and are already competent in the step-by-step logic of adjustment. Here, our criticism is about the fact that Mol’s conception of care does not discuss the difficulties that are immanent to this step-by-step logic of adjustment, such as doctors’ and patients’ hidden agendas, scepticism or denial, which also implies the knowledge of their common human manner of reacting to vulnerability. In other terms, Mol does not consider the relational difficulties linked to fundamental disagreements, resistance to medicalisation, or people’s unconscious attachments to personal and normative opinions, claims or visions. Consequently, the pragmatic logic of care, as described by Mol, does not help to put words to patients’ and doctors’ anthropological and existential difficulties or vulnerability.

According to Mol’s logic of care, everything seems to happen as if the issues and the agendas were known a priori, as if what really matters to both patients and doctors was inter-subjectively given and already acknowledged as if the issues were already shaped and known. Furthermore, as described by Mol, the logic of care ignores the existential vulnerability of physicians and patients. In a word, it lacks a serious discussion of the ordinary moral vulnerability of care. Here again, our discussion of Mol’s logic of care echoes GPs’ implicit theory of care, which refers to patients’ and doctors’ vulnerabilities mostly in terms of technologies used to manage clinical uncertainties rather than in terms of anthropological and existential difficulties of knowing and acknowledging what matters, what make sense and what the value of medicalisation really is.

## Conclusion: the taboo of vulnerability in medicine

This paper showed how GPs ways of speaking about (over-)medicalisation leave the issues of vulnerability in the shadows. We identified five defensive paradigms in GPs’ attitudes towards (over-)medicalisation. Each paradigm (underlying social factors, preventing medicalisation, managing uncertainties, sharing medical decision-making and thinking about care as a rationale) was brought up in our focus groups, echoing contemporary political debates in public health. These non-exhaustive paradigms help sketch the outline of what we would like to call *ordinary defensive medicine*. But our findings beg the question, defensive paradigms against what? The existence of defences reveals medicine’s taboo of vulnerability. We suggested throughout this paper that the feelings of discomfort, guilt and being pressured from all sides do not come from “outside”. They come from the concrete realities and ordinary normativity (e.g. their attachment to medical norms, to predefined ways of caring and living and to their resistance to medicalise) that animate GPs’ encounters with their patients. Nevertheless, GPs never speak about the idea that (over-)medicalisation is an issue immanent to encounters with their patients, nor that it could be discussed and co-constructed with them. Accordingly, by keeping them at a “rational” distance, GPs’ attitudes towards (over-)medicalisation prevent them from thinking and speaking about their role and involvement in therapeutic processes. And this very distance is what led us to speak about some of their attitudes as being defensive. Here, the issue is political because it is a part of avoiding thinking, speaking and acknowledging the ethical and moral vulnerabilities in medicine. As our two introductory examples argued, this provides an opportunity to rediscover the vulnerability of medical relationships and therapeutic processes—the encounter between at least two vulnerable human beings who do not want to, or have some difficulties, sharing and acknowledging their existential interdependency. And this encourages us to ask about the kind of ethics and politics of vulnerability we would like to promote within our health care systems and our social environments. Do we want ethics and politics that comfort us and maintain our deeper vulnerabilities at a rational distance or do we want them to acknowledge the fact that solutions and meanings can be imagined, improvised and co-formed with others? In this view, there are two possible postures toward vulnerability: working with it or working against it. This leads us to yet another ethical and political question: how much violence arises when people adopt defensive postures and deny vulnerability within medical care?

## Data Availability

Data are available at the Department of Family Medicine, General Medicine and Public Health Centre, University of Lausanne.

## Abbreviations

EBM: Evidence-based medicine
GP: General practitioner
(over-)medicalisation: Medicalisation and over-medicalisation
CME: Continuing medical education
